# Specifically targeting toxic forms of tau modulates AD pathologies and inflammageing in very old triple‐transgenic mice

**DOI:** 10.1002/alz.087682

**Published:** 2025-01-03

**Authors:** Alessia Sciortino, Sudipta Senapati, Fadhl Al‐Shaebi, Madison Samples, Rakez Kayed

**Affiliations:** ^1^ University of Texas Medical Branch, Galveston, TX USA

## Abstract

**Background:**

Rodent models have been proved pivotal in Alzheimer’s disease (AD) research. Nevertheless, the use of models that only recapitulate one aspect of AD neuropathology, and of early time points that might be excluding important features such as age‐dependent inflammation and senescence, could hinder the development of effective AD therapeutics. Several tau immunotherapies are currently undergoing clinical trial. Misfolding and accumulation of tau have been shown to correlate with Alzheimer’s disease (AD) progression and dementia stage. Numerous studies showed that tau removal is able to slow down AD pathology propagation and cognitive deterioration in different animal models.

**Method:**

We developed in‐house anti‐tau antibodies that target specific toxic tau conformations and evaluated immunotherapy impact on AD‐like pathology in very old triple‐transgenic AD mice (3xTg AD), in order to capture age‐dependent aspects often overlooked in other studies. Twenty‐three months old 3xTg AD mice, that reproduce both amyloid and tau pathology, and other cellular changes associated with AD, were injected in the tail vein with 120µg of anti‐toxic tau antibody or IgG isotype control. Behavioral tests to evaluate motor and cognitive performance were carried out one week post injection. Immunohistochemistry, immunofluorescence and western blot techniques were used to investigate brain neuropathology following toxic tau removal.

**Result:**

Treatment with conformational antibodies targeting toxic forms of tau modulated the extent of AD pathology, in the form of tau and amyloid deposition, in hippocampus and cortex. Furthermore, immunotherapy modified markers of age‐related pathology, suggesting an interplay of toxic tau with the ageing process i.e. inflammation and senescence.

**Conclusion:**

Our study contributes to the investigation of selective tau immunotherapy approaches for AD and tauopathies. Our results suggest that specifically targeting toxic forms of tau modulates AD pathology beyond pathological protein aggregation and could have beneficial effects even at later disease stages.

